# Application of a photogrammetric kinematic model for prediction of lung volumes in adolescents: a pilot study

**DOI:** 10.1186/1475-925X-13-21

**Published:** 2014-02-27

**Authors:** Wagner L Ripka, Leandra Ulbricht, Pedro M Gewehr

**Affiliations:** 1 Graduate Program in Electrical Engineering and Computer Science, Federal University of Technology-Paraná, Av. Sete de Setembro, 3165 Curitiba, Brazil; 2 Graduate Program in Biomedical Engineering, Federal University of Technology-Paraná, Av. Sete de Setembro, 3165 Curitiba, Brazil

**Keywords:** Photogrammetry, Spirometry, Adolescent

## Abstract

**Background:**

There are several ways to measure the respiratory system, among them inductance plethysmography and three-dimensional kinematic analysis, methods of high cost and difficult transportability. The objective of this study was to correlate respiratory volumes obtained by spirometry standard equipment with a biomechanical model photogrammetric analysis of adolescents.

**Methods:**

We evaluated 50 subjects of both genders, aged between 14 and 17 years old, excluding those with respiratory obstruction or restriction. Stickers with markers, there was a five-point mapping for anatomical modeling and photogrammetry, with each evaluated in supine position, was sought to test the Forced Vital Capacity (FVC). The test was filmed and repeated three times. Images of the films were extracted for the moment of maximum exhalation and inhalation of proof with better breathing. With the use of a commercial software, defined the respiratory volumes to the thorax and abdomen.

**Results:**

The photogrammetric analysis has found values strongly correlated with the spirometric measurements of FVC (0.812), forced expiratory volume in one second (FEV_1_ – 0.708), Peak Expiratory Flow (PEF – 0.762) in addition to post test performed Inspiration (IP- 0.816). There was a higher ventilatory mobility for boys than girls for Lower Chest and Lower and Upper Abdomen. It was possible to reach a regression R^2^ = 0.866 for proof of FVC and R^2^ = 0.776 for IP with the use of photogrammetry, presenting a standard error of 0.353 and 0.451, respectively.

**Conclusions:**

Photogrammetry can be used to study thoracoabdominal movements by applying analytical two-dimensional and three-dimensional images acquired using a video camera being, applicable and reproducible*.*

## Background

The respiratory performance is the result of a complex set of organs, muscles and bone structures that work simultaneously to generate the phenomenon of ventilation, vital for humans [1]. The measures of ventilation reflects physiological and functional aspects, with potential use in the clinical, epidemiological and sports areas [2].

Technologies that aim to measure and evaluate respiratory performance are fundamental for health professionals' decision making [3]. Thus, tools that produce consistent values, with objectivity, reproducibility and relevance, are designed to facilitate such work [4].

Most of the available methods for evaluation volumes, capacities, limitations and breathing patterns in an efficient and non-invasive way are, to Brazilian health area, expensive and often difficult to transport, and it requires trained technicians to operate [5-7]. These limitations restrict pulmonary function test and measures of ventilation to large laboratories, preventing their popularization in many places, such as schools, basic health units and clubs [8].

Considering the reality of Brazilian public health system, there is a need to develop low cost tools and methodologies for respiratory measures that provide consistent and reproducible results. Photogrammetry, a process dating from 19th century, enables obtaining quantitative information about a system that will be evaluated by inserting metric dimensions into images. Its association with static and kinematic measures has indicated satisfactory results [9-12], being considered as a new way for the aggregation of high-level evidence in the evaluation of form and quality of movement [13-15].

This process, incorporated to the respiratory measures, showed good results in two-dimensional respiratory analysis, proving to be a reliable, reproducible and affordable method [8,16]. However, studies with adolescents, such as three-dimensional ventilation simulations, show that there are still gaps to be filled. Therefore, the objective of this study was to test the applicability of a biomechanical model of three-dimensional (3D) photogrammetric analysis for predicting lung volumes in adolescents, in comparison to spirometry, the standard method for respiratory measures.

## Methods

An exploratory-descriptive study was carried out with a sample of 50 individuals (19 boys and 21 girls), aged between 14 and 17 years old. The assessments included anthropometry, spirometric measurements and photogrammetric analysis. Parents or guardians of all participants signed a formal letter of consent. This study was approved by the Ethics Committee of the UFPR under protocol no. 01655012.6.0000.0102.

Body mass (kg), height (m), body mass index (BMI - kg/m2) and thorax length (cm) were collected for the anthropometric assessment.

Body mass was measured by using a 100 g resolution mechanical scale (Filizola™). Height was assessed by using a stadiometer (0.1 cm resolution), fixed and coupled to the scale. Data collection followed recommendations described in the literature [17].

BMI enables classification of participants as underweight, normal, overweight and obese. In order to measure thorax length, participants stood up with their arms away from their bodies. An anthropometric caliper (WCS model; 0.1 mm resolution) was positioned in line with the xiphoid process of each participant, who were asked to take a deep breath during the measurement.

The respiratory evaluation was a selection criterion for the study. The procedures followed recommendations of the Brazilian Society of Pulmonology and Phthisiology [18]. Each participant, after previous training, was asked to perform three forced vital capacity (FVC) tests using a bi-directional digital spirometer (CareFusion – Microloop model) with accuracy of 10 ml for volume and 0.03 l/s ± 3% for flow.

In addition to FVC values, the maneuver allowed to obtain the peak expiratory flow (PEF), the forced expiratory volume in one second (FEV1), deep inspiration (DI) and the Tiffeneau index (FEV1/FVC). Participants with a Tiffeneau index below 80% were excluded, since this is an indicative of respiratory obstruction or restriction [18].

Seven adolescents had a Tiffeneau index lower than 80%, and three had possible restriction due to low FVC. Therefore, 40 individuals were analyzed for the treatment of images.

Participants with a Tiffeneau index greater than 80% underwent a new respiratory assessment that included three new tests in dorsal decubitus position. These three maneuvers were filmed, and the measurement with the best expiratory value was used in the photogrammetric analysis.

**Figure 1:**
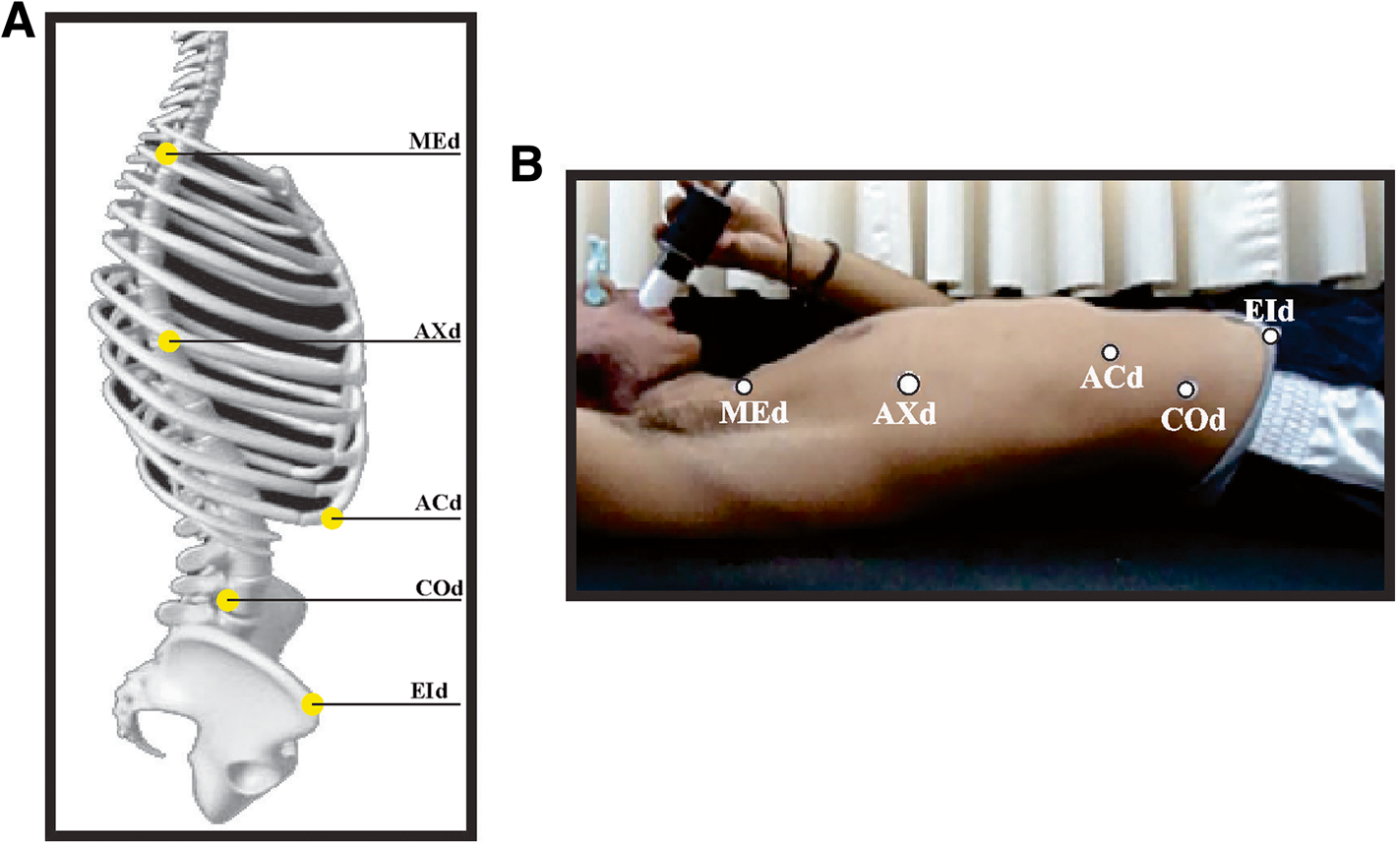
**Theoretical and practical representation of the markers. A)** Theoretical representation of the markers on the thoracoabdominal region. Projections of the umbilicus (COd), the inferior angle of the 10th rib (ACd), the manubrium sterni (MEd), the xiphoid process (AXd) and the right anterior superior iliac spine (EId). **B)** Practical representation of the markers position with the subject in the dorsal decubitus position.

A Sony camcorder HDX-100 supported on a 1.50 m tripod and placed 2 m away from the participant was used. Adhesive markers (PIMACO®) with known diameter (13 mm) were placed on the participants on the projections of the umbilicus (COd), the inferior angle of the 10th rib (ACd), the manubrium sterni (MEd), the xiphoid process (AXd) and the right anterior superior iliac spine (EId) (Figure 1A and B). The determination of anatomic location was given by following the methodology of palpatory anatomy by Tixa [19].

**Figure 2:**
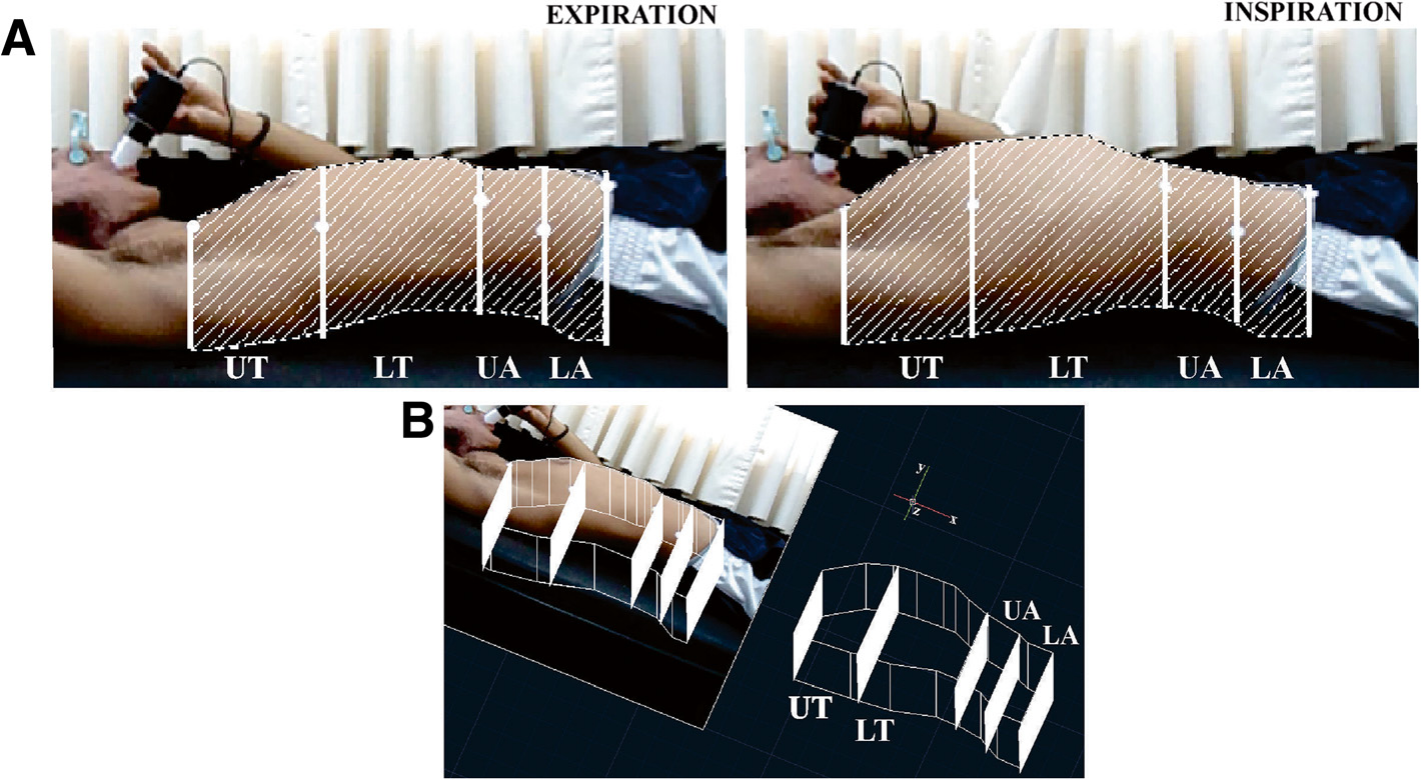
**Representations of the photogrammetric model. A)** Representation of the photogrammetric model for maximal inspiration and expiration. **B)** Photogrammetric model of three-dimensional extrapolation from two-dimensional images. In both images, the thoracoabdominal section shows the Upper Thorax (UT), Upper Abdomen (UA), Lower Thorax (LT) and Lower Abdomen (LA). The sets UT-LT and UA-LA form the Total Thorax Area (TT) and Total Abdominal Area (TA), respectively.

The Windows Movie Maker software was used to extract the frames related to the moments of maximum expiration and inspiration. These images were transferred to AutoCad® version 2012 for three-dimensional extrapolation (Figure 2) and obtaining thoracic and abdominal volumes. All calculations were carried out after the dimension correction of each image, based on the adhesive marker with known diameter. The 3D extrapolation was made from the insertion of the thoracic length in the 2D image, through the extrusion tool available in the software.

The results were initially analyzed by position and dispersion measurements (mean and standard deviation). Then, the Pearson's correlation test was applied to verify the hypothesis between respiratory volumes and surface thoracoabdominal displacement. The multiple regression test was applied to structure equations for FVC and DI prediction. Finally, the Bland-Altman test was used to verify the agreement between the new method applied and the standard spirometric procedure. The limits were defined with the mean bias ± SD 1.96 [20]. Statistical significance was set at p <0.05.

## Results

Table 1 shows mean, minimum and maximum values of the anthropometric, respiratory and photogrammetric measures. Age (years), body mass (kg), height (m), BMI (kg.m-2) and thorax length (cm) are the variables for the anthropometric values. Forced expiratory volume in one second (FEV1) (l), forced vital capacity (FVC) (l), peak expiratory flow (PEF) (l/s) and deep inspiration (DI) were part of the pulmonary function assessment (l). The photogrammetric analysis relates to the volumes of Upper Thorax, Lower Thorax and Total Thorax, Upper Abdomen, Lower Abdomen and Total Abdomen, and Thoracoabdominal region. The volumetric values were described for inspiration and expiration.

**Table 1:** Sample table title

**Variables**	**Mean**	**Standard deviation**	**Minimum**	**Maximum**
**Anthropometric values**				
Age (years)	15.4	1.0	14.0	17.0
Body mass (kg)	61.3	12.3	43.5	87.0
Height (m)	1.66	0.1	1.52	1.87
BMI (kg.m^-2^)	22.1	3.5	17.6	32.3
Thorax (cm)	25.5	1.9	21.5	29.5
**Pulmonary function**				
FEV1 (l)	3.22	0.82	1.77	5.16
FVC (l)	3.87	0.91	2.39	5.81
PEF (l/s)	6.50	2.41	2.84	12.15
DI (l)	3.59	0.91	2.05	5.40
**Photogrammetric analysis (expiration)**				
Upper thorax (l)	7.32	2.44	4.30	15.22
Lower thorax (l)	5.90	2.21	2.51	11.12
Total thorax (l)	13.21	4.14	7.82	26.34
Upper abdomen (l)	2.56	0.87	0.91	5.19
Lower abdomen (l)	3.56	1.56	0.74	8.41
Total abdomen (l)	6.12	2.2	2.53	13.18
Thoracoabdominal (l)	19.33	6.18	10.36	39.52
**Photogrammetric analysis (inspiration)**				
Upper thorax (l)	8.41	2.62	4.93	15.17
Lower thorax (l)	7.65	3.25	3.05	17.04
Total thorax (l)	16.06	5.24	9.75	30.79
Upper abdomen (l)	3.16	1.19	1.13	6.87
Lower abdomen (l)	3.86	1.83	0.87	9.39
Total abdomen (l)	7.02	2.75	2.66	15.69
Thoracoabdominal (l)	23.08	7.77	12.41	46.31

Mean, standard deviation, minimum and maximum values for age, weight, height, BMI, thorax length, forced expiratory volume in one second (l), forced vital capacity (l), peak expiratory flow (l/s), deep inspiration (l) (DI) and photogrammetric analysis for expiration and inspiration tests.

All participants showed normal BMIs and spirometric values recommended by the Brazilian Society of Pulmonology and Phthisiology [18]. Analyzing the volumetric variability between inspiration and expiration, the values 14.9%, 29.7% and 21.6% were obtained for UT, LT and TT, respectively, whereas 23.4% , 8.4% and 14.7% were obtained for UA, LA and TA, respectively. These values indicate greater recruitment of the UT region followed by the UA region. In the analysis of inspiration, the proportion of TT and TA use in relation to the thoracoabdominal area was also evaluated: 69.6% for TT against 30.4% for ABT was observed.

Table 2 shows the correlation values between the pulmonary function and the photogrammetric results. Correlations between all compartments evaluated by photogrammetry with the respiratory variables can be noted, and the highest values are for FVC and DI with the Total Volumetric Mobility. Volumetric Mobility values were obtained through the difference between the inspired and expired thoracoabdominal volumes, both calculated in the 3D extrapolation.

**Table 2:** Another sample table title

	**FEV1**	**FVC**	**PEF**	**DI**
**Inspiration**				
Upper thorax (l)	0.625	0.680	0.658	0.701
Lower thorax (l)	0.670	0.709	0.715	0.660
Total thorax (l)	0.728	0.780	0.773	0.760
Upper abdomen (l)	0.480	0.513	0.571	0.549
Lower abdomen (l)	0.610	0.642	0.625	0.636
Total abdomen (l)	0.616	0.664	0.665	0.663
Thoracoabdominal (l)	0.708	0.761	0.756	0.747
**Expiration**				
Upper thorax (l)	0.586	0.628	0.635	0.646
Lower thorax (l)	0.603	0.613	0.604	0.550
Total thorax (l)	0.668	0.698	0.697	0.674
Upper abdomen (l)	0.398	0.470	0.519	0.474
Lower abdomen (l)	0.613	0.619	0.635	0.604
Total abdomen (l)	0.594	0.627	0.658	0.618
Thoracoabdominal (l)	0.659	0.691	0.701	0.672
Total volumetric mobility	0.708	0.812	0.762	0.816

Correlation between the inspired and expired thoracoabdominal values obtained by photogrammetry and respiratory volumes obtained by spirometry.

Finally, photogrammetric analysis was employed for the prediction of the values obtained with a spirometer, through linear regression. The model for FVC prediction had correlation R = 0.931 (R^2^ = 0.866), standard deviation = 0.353 and p <0.05 (Equation 1). It was also possible to predict the DI value with correlation R = 0.881 (R^2^ = 0.815), standard deviation = 0.451 and p <0.05 (Equation 2). Height (m), thorax length (cm) and total volumetric mobility (l) were used in equations (1) and (2):

(1) $$\begin{array}{l} \mathrm{FVC}=‒8.572+5.108\;\times \;\mathrm{Height}+0.138\;\times \;\mathrm{Thorax}+0.120\;\\ \;\times \;\mathrm{Total}\;\mathrm{Volumetric}\;\mathrm{Mobility} \end{array}$$

(2) $$\begin{array}{l} \mathrm{DI}=‒6.373+3.751\;\times \;\mathrm{Height}+0.122\;\times \;\mathrm{Thorax}+0.163\;\\ \;\times \mathrm{Total}\;\mathrm{Volumetric}\;\mathrm{Mobility} \end{array}$$

**Figure 3:**
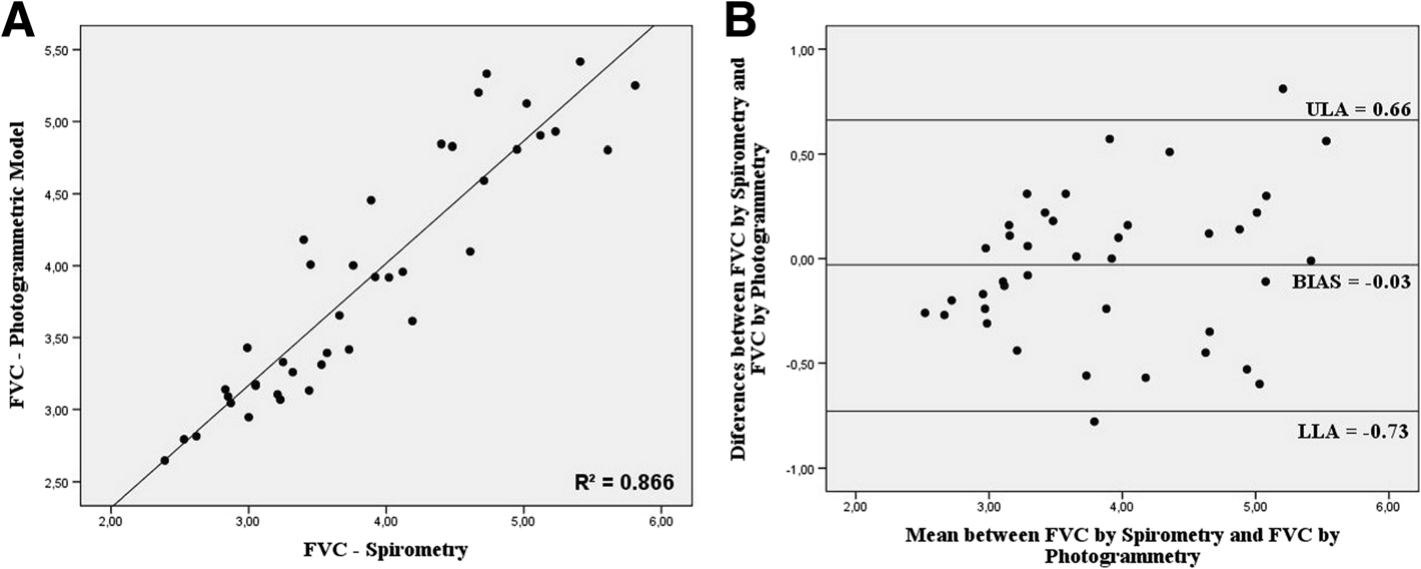
**Bland-Altman plot by photogrammetric and FVC. A)** Plot for the values predicted by photogrammetric analysis and for FVC by spirometry. **B)** Bland-Altman analysis of agreement. ULA = Upper Limit of Agreement; LLA = Lower Limit of Agreement.

**Figure 4:**
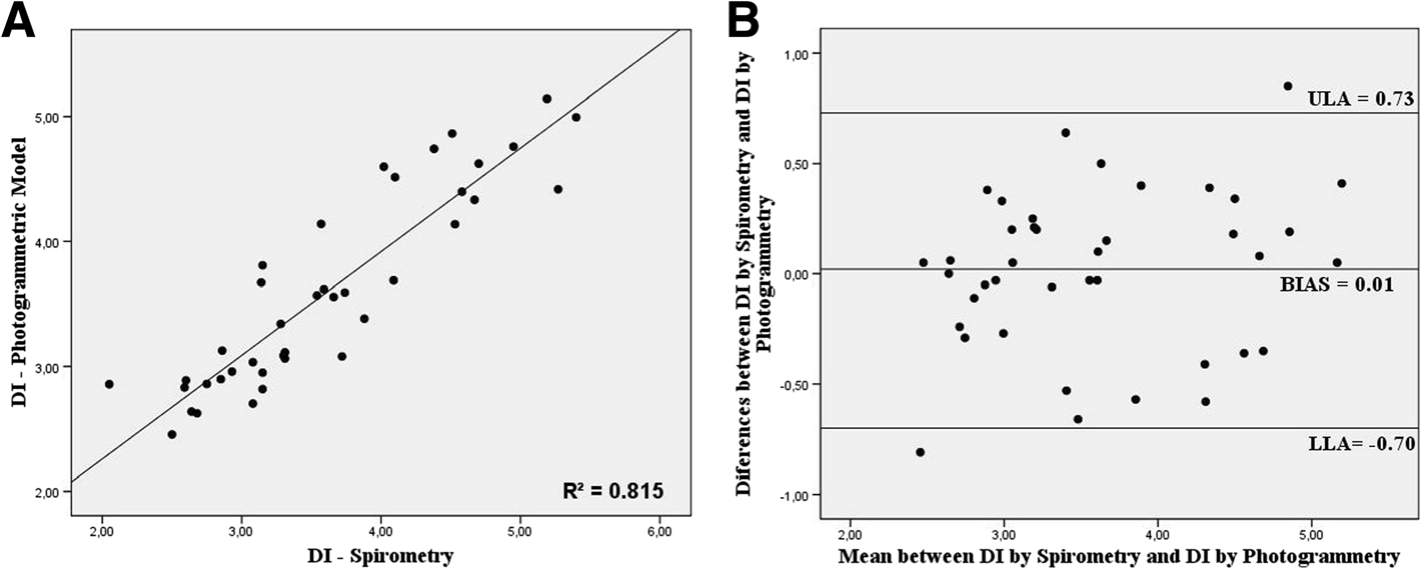
**Bland-Altman plot by photogrammetric and DI. A)** Plot for the values predicted by photogrammetric analysis and for DI by spirometry. **B)** Bland-Altman analysis of agreement. ULA: Upper Limit of Agreement; LLA = Lower Limit of Agreement.

A method of agreement was tested with the Bland-Altman plot, as shown in Figure 3 for FVC and in Figure 4 for DI. There was no significant bias for any of the predictions.

## Discussion

All participants were considered as normal through spirometry analysis, with average values of 3.22 ± 0.82 l for FEV1, 3.87 ± 0.91 l for FVC and 6.50 ± 2.41 l/s for PEF. Spirometry was used for comparing photogrammetric data, since spirometry test is considered an important tool for the measures, quantification and diagnostic follow-up of pulmonary function [21,22].

The methodology applied in this study allowed the analysis of the contribution of the thoracic and abdominal compartments in the inspiration process of the participants, showing greater expansibility for the LT and UA regions. The ratios of 69.6% for TT and 30.4% for TA are similar to those found in the literature [8,14]. Mechanically interconnected by the diaphragm, compartments TX and AB work synchronously to capture respiratory gases, yet possess independent movement [8,23,24], allowing greater expansion of one region over another.

In this study, FVC and DI predictions showed a good correlation with spirometry, since respiratory capacities suffer numerous technical impacts, including: training to perform the tests and biological individualities [25], observing values of R^2^ = 0.866 and R^2^ = 0.815, respectively.

Prediction equations for respiratory values are found in the literature to establish predicted values for variables such as FVC, FEV1 and FVC however, variability can reach up to 20% of the actual value, especially for FVC [26-28]. The maximum variability, represented by the standard deviation, resulted 7.9% for FVC and 8.2% for DI. Regression tests are seen in the respiratory prediction equations, which has height as the primary independent variable [29,30]. In this study, the inclusion of thoracoabdominal mobility values computed by 3D photogrammetry, as well as the inclusion of the thoracic length, minimized the errors inherent in predicting lung volumes [31].

Quantifying the respiration in its cycles is important because it allows the analysis of respiratory patterns that may represent efficiency or deficiency in energy demand. About 3 to 5% of the energy consumed by the body occurs in the quiet or basal ventilation [32], and this value can be even higher (30%) in individuals with some degree of obstruction or restriction [1].

The quantification of respiratory movements based on the variations in lung volumes, through the displacement of thoracoabdominal structures and associated with photographic images, is a facilitating strategy to quantitative identification of imbalances in the respiratory system. Moreover, this method has been proven effective, of easy applicability and transportability [8,12,14,31].

The spirometry is considered a low-cost method in developed countries, however the reality of public health in developing nations shows otherwise. In Brazil, a country of this study, it is the duty of the state to secure health for all residents, however the lack of equipment and lack of maintenance are typical themes in news. Data show that the Brazilian public health system provides 1.9 device per million inhabitants and estimates that 30% of all technology park is not used properly or is abandoned [33], reinforcing the need for creation of new methodologies.

Although the results found in this work, 3D photogrammetric analysis lacks comparison to validated laboratory methods used to study respiratory kinematics, such as inductance plethysmography and analysis of three-dimensional images.

## Conclusions

The equations developed based on the suggested process for predicting FVC and DI showed high correlation, and their agreement was tested by the Bland-Altman plot.

Respiratory analysis through images is a relatively simple and fast process; it involves easily transportable tools, allowing the application of respiratory movements tests in several places, including schools, gyms and clubs. In addition, photogrammetric studies in adolescents represent an innovation in the literature, as previous studies in respiratory imaging analysis have been restricted to children and adults.

This research achieved the proposed objectives; however, a photogrammetric analysis during effortless ventilation maneuvers is suggested for future studies, in order to test the sensitivity of the method in small thoracoabdominal displacements. There is also the need for application of the method in different populations, such as individuals evaluated in different positions, since this is a variable that changes respiratory values.

## Competing interests

The authors declare non-financials competing interests.

## Authors' contributions

WLR participated in the design of the study, performed the statistical analysis and wrote the manuscript; LU participated in the mythology and study review; PMG participated in its design and coordination. All authors read and approved the final manuscript.
